# Counteraction of Myocardial Ferritin Heavy Chain Deficiency by Heme Oxygenase-1

**DOI:** 10.3390/ijms23158300

**Published:** 2022-07-27

**Authors:** Sarah E. Machado, Daryll Spangler, Delores A. Stacks, Victor Darley-Usmar, Gloria A. Benavides, Min Xie, József Balla, Abolfazl Zarjou

**Affiliations:** 1 Division of Nephrology, Department of Medicine, University of Alabama at Birmingham, Birmingham, AL 35294, USA; smachado@uab.edu (S.E.M.); daryll.r.spangler@gmail.com (D.S.); delorez@uab.edu (D.A.S.); 2 Mitochondrial Medicine Laboratory, Department of Pathology, University of Alabama at Birmingham, Birmingham, AL 35294, USA; vdarleyusmar@uabmc.edu (V.D.-U.); gloriabenavides@uabmc.edu (G.A.B.); 3 Division of Cardiovascular Disease, Department of Medicine, University of Alabama at Birmingham, Birmingham, AL 35233, USA; mxie@uabmc.edu; 4 ELKH-UD Vascular Pathophysiology Research Group 11003, Division of Nephrology, Department of Internal Medicine, Faculty of Medicine, University of Debrecen, H-4032 Debrecen, Hungary; balla@belklinika.com

**Keywords:** heme oxygenase-1, ferroptosis, ferritin, Slc7a11

## Abstract

Given the abundance of heme proteins (cytochromes) in the mitochondrion, it is evident that a meticulously orchestrated iron metabolism is essential for cardiac health. Here, we examined the functional significance of myocardial ferritin heavy chain (FtH) in a model of acute myocardial infarction. We report that FtH deletion did not alter either the mitochondrial regulatory and surveillance pathways (fission and fusion) or mitochondrial bioenergetics in response to injury. Furthermore, deletion of myocardial FtH did not affect cardiac function, assessed by measurement of left ventricular ejection fraction, on days 1, 7, and 21 post injury. To identify the modulated pathways providing cardiomyocyte protection coincident with FtH deletion, we performed unbiased transcriptomic analysis. We found that following injury, FtH deletion was associated with upregulation of several genes with anti-ferroptotic properties, including heme oxygenase-1 (HO-1) and the cystine/glutamate anti-porter (Slc7a11). These results suggested that HO-1 overexpression mitigates ferroptosis via upregulation of Slc7a11. Indeed, using transgenic mice with HO-1 overexpression, we demonstrate that overexpressed HO-1 is coupled with increased Slc7a11 expression. In conclusion, we demonstrate that following injury, myocardial FtH deletion leads to a compensatory upregulation in a number of anti-ferroptotic genes, including HO-1. Such HO-1 induction leads to overexpression of Slc7a11 and protects the heart against ischemia-reperfusion-mediated ferroptosis, preserves mitochondrial function, and overall function of the myocardium.

## 1. Introduction

Despite scientific and technical progress in risk prediction, diagnostics, prognostication and therapeutic approaches, acute myocardial infarction (MI) remains a leading cause of morbidity and mortality worldwide [1,2,3]. It is estimated that MI results in over 7 million deaths every year globally [2,3]. In the United States, estimates suggest that an MI occurs every 40 s [3]. Such staggering statistics highlight the desperate need to improve mortality, morbidity, and health care expenditures related to ischemic heart disease.

Early coronary artery reperfusion strategies significantly improve outcomes for patients with ST-segment elevation [4]. Nevertheless, animal models demonstrate that approximately half of the final size of a myocardial infarct is due to reperfusion injury [5,6,7]. During the reperfusion phase, the myocardium is exposed to rapid metabolic and biochemical alterations that include substantial production of ROS, mitochondrial re-energization, marked elevation of intracellular calcium levels, and instigation of a profound inflammatory response [8]. These dynamic modulations interact and result in mitochondrial dysfunction, ATP depletion, damage to vital intracellular homeostatic machinery with ensuing myocardial hypercontracture and eventual cardiomyocyte death [4,8]. Several of the aforementioned perturbations are now established components of a distinct form of programmed cell death, namely ferroptosis [9]. Importantly, a growing body of evidence suggests that ferroptosis is a central pathophysiologic pathway in several cardiovascular diseases, including MI [10,11,12,13]. 

Cardiac mitochondrial integrity and function is heavily dependent on iron homeostasis but if unregulated can promote ROS formation with ensuing cellular and tissue injury through various mechanisms, including ferroptosis [12]. Indeed, iron metabolism and lipid peroxidation are increasingly recognized as fundamental regulators of ferroptosis. The evolutionary solution to this challenge involves a large nanocage capable of safely storing up 4500 atoms, namely ferritin. Intracellular ferritin is composed of heavy (FtH) and light (FtL). FtH possesses ferroxidase activity converting Fe^2+^ to Fe^3+^, facilitating its safe storage within the sphere [14,15]. The paramount importance of FtH is further highlighted by early embryonic lethality in mice with global deletion of FtH [16]. The proportional contribution of FtH and FtL to the hollow spherical shell depends on the tissue and developmental stage of the organism. Notably, brain and heart ferritin is primarily composed of FtH chains, while the liver and spleen mainly possess FtL [17]. We and others have validated the beneficial functional significance of FtH in different diseases models [18,19,20,21,22]. However, the full extent of FtH contribution to the myocardial response to injury and corresponding molecular pathways remain to be fully elucidated. Given the intricate relation among iron/heme metabolism, mitochondrial activity, and ferroptosis, we sought to investigate whether myocardial deletion of FtH leads to heightened injury and disruption in mitochondrial biogenesis in a model of ischemia-reperfusion (IR)-mediated injury. We postulated that myocardial FtH deletion would result in heightened disruption in mitochondrial biogenesis, ferroptosis, and ensuing cardiac function decrement. To address these questions, we generated mice with myocardial FtH deficiency (FtH^SM22Δ/Δ^) to examine the role of FtH in MI and its impact on mitochondrial surveillance processes and biogenesis.

## 2. Results

### 2.1. Characterization of FtH^SM22Δ/Δ^ Mice

**Figure 1:**
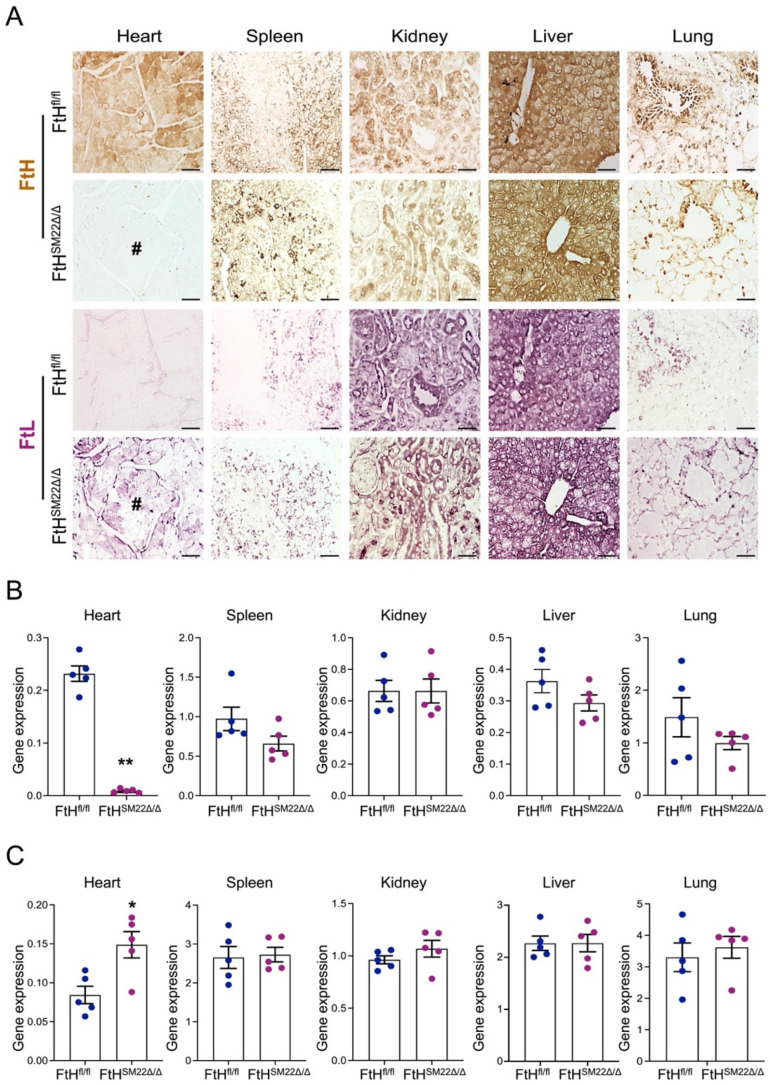
Establishment of FtH^SM22Δ/Δ^ mice. (**A**) Immunohistochemical staining of serial sections of heart, spleen, kidney, liver, lung tissues from FtH^fl/fl^ and FtH^SM22Δ/Δ^ mice illustrating FtH (brown) and FtL (pink) expression. Scale bar = 100 μm. # denotes lack of FtH expression in cardiomyocytes concomitant with FtL overexpression in corresponding serial section. n = 5/genotype. (**B**,**C**) Various organs from FtH^fl/fl^ and FtH^SM22Δ/Δ^ were harvested and mRNA levels of FtH (**B**) and FtL (**C**) were examined using RT-PCR analysis. Results were normalized to GAPDH and presented as means ± SEM. * *p* < 0.05 and ** *p* < 0.01. n = 5/genotype.

To investigate the relationship between myocardial iron homeostasis and MI, and the role of FtH in this context, we generated transgenic mice with targeted deletion of FtH in cardiomyocytes. FtH deletion was achieved by crossing FtH^fl/fl^ mice with SM22-Cre mice that express the Cre protein in vascular smooth muscle cells and myocardium during the embryonic period [23]. FtH^SM22Δ/Δ^ mice were born at the expected Mendelian ratio, were viable and fertile, and did not manifest any apparent abnormalities during 6 months of observation. FtH^fl/fl^ mice—homozygous for the floxed allele with no changes in FtH expression—were used as controls. The recombination event by Cre was confirmed by PCR using genomic DNA with specific primers. Using immunohistochemistry, we examined expression of FtH protein in five major organs, namely, heart, spleen, kidney, liver, and lung (Figure 1A). 

As expected, we demonstrate a marked reduction in myocardial FtH expression in FtH^SM22Δ/Δ^ mice when compared to FtH^fl/fl^ littermates. In contrast, no noticeable differences in terms of FtH expression were observed in other major organs (Figure 1A). Next, we analyzed expression of FtL in the aforementioned organs. As illustrated in Figure 1A, we found modest myocardial expression of FtL in FtH^fl/fl^ mice, while such expression was noticeably higher in hearts of FtH^SM22Δ/Δ^ mice. These findings corroborate previous reports that identify the FtH chain as the main contributor to the ferritin shell in myocardium [24], and highlight the compensatory increment of FtL in the absence of FtH expression [25]. Deletion of FtH in myocardium was further validated by mRNA expression. Our results in Figure 1B reveal a significant reduction in FtH mRNA levels in the myocardium of FtH^SM22Δ/Δ^ mice. Moreover, we confirm the compensatory increase in myocardial FtL mRNA in the absence of FtH expression (Figure 1C). Similar to immunohistochemistry results, no significant alteration was noted in FtH, or FtL mRNA in other organs.

### 2.2. Impact of FtH Deletion on Mitochondrial Surveillance Processes and Bioenergetics following IR

**Figure 2:**
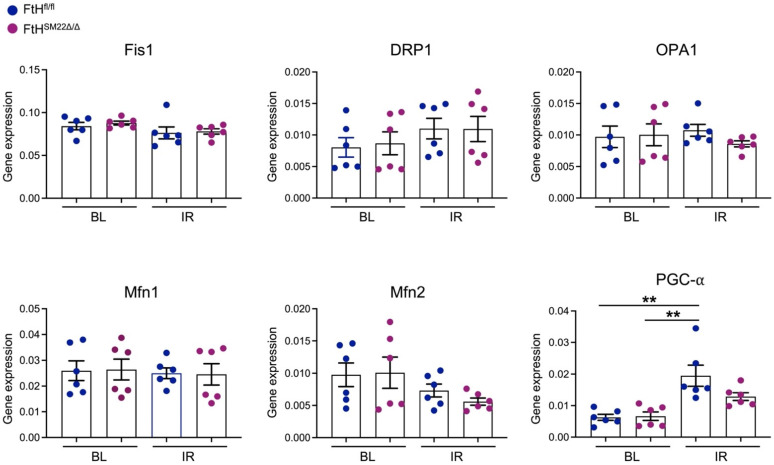
FtH deletion does not alter expression of genes involved in mitochondrial fission and fusion processes following ischemia-reperfusion (IR). RNA from the left ventricle of FtH^fl/fl^ and FtH^SM22Δ/Δ^ mice under baseline (BL) conditions and the ischemic region at 3 h post IR was isolated and expression levels of fission and fusion genes as well as PGC-1α were examined using RT-PCR analysis. Results were normalized to GAPDH and presented as means ± SEM. n = 5/condition/genotype. ** *p* < 0.01.

Mitochondria are highly dynamic organelles that continually change their shape via processes known and fusion and fission. These processes are known to affect the course of IR-mediated myocardial injury. Given the central role of iron in mitochondrial health and disease, we asked whether FtH deletion may impact the fission and fusion processes following IR. To address this question, aged-matched, male FtH^fl/fl^ and FtH^SM22Δ/Δ^ littermates were subjected to myocardial IR via a reversible ligation of left anterior descending coronary branch for 45 min. At three hours post reperfusion, the ischemic region was carefully dissected, and total RNA was isolated for further analysis. We examined the expression levels of fission (Fis1, Drp1) and fusion (Opa1, Mfn1, Mfn2) related genes to investigate how myocardial FtH deletion may affect their expression following IR-mediated injury. As illustrated in Figure 2, we did not find any significant differences amongst the genotypes under baseline (BL) conditions or following IR. 

**Figure 3:**
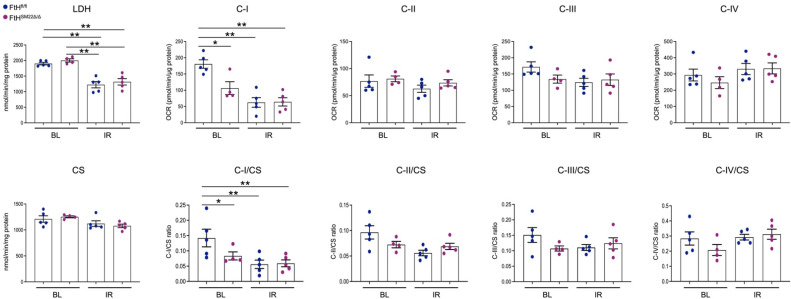
Evaluation of mitochondrial complex, lactate dehydrogenase (LDH), and citrate synthase (CS) activity in frozen heart lysates from BL and IR conditions in FtH^fl/fl^ and FtH^SM22Δ/Δ^ mice. Protein was isolated from the left ventricle under BL conditions and the ischemic region at 3 h following IR from FtH^fl/fl^ and FtH^SM22Δ/Δ^ frozen hearts. Mitochondrial complex activity including NADH (complex I), succinate (complex II), duroquinol (complex III), or tetamethylphenylenediamine (complex IV) was measured in heart lysates from BL and IR conditions in FtH^fl/fl^ and FtH^SM22Δ/Δ^ mice and oxygen consumption rate (OCR) was expressed as pmol/min/μg protein. LDH and CS activities are expressed as nmol/min/mg protein. Graphs depict cumulative quantification of data; n = 4–5. Data are presented as means ± SEM. * *p* < 0.05 and ** *p* < 0.01.

Furthermore, we also evaluated the mRNA levels of peroxisome proliferator-activated receptor-gamma coactivator (PGC-1α), a transcriptional coactivator with central and key regulation of cellular energy metabolism [26]. We found that PGC-1α was significantly upregulated in hearts of FtH^fl/fl^ mice following IR when compared to BL conditions. Conversely, while there was a modest increase in PGC-1α in FtH^SM22Δ/Δ^ hearts following IR, such increment was not statistically significant when comparing both genotypes post injury, *p* = 0.113 (Figure 2). Based on these findings we postulated that lack of PGC-1α may negatively affect mitochondrial biogenesis and energy production. To test this hypothesis, we measured the citrate synthase and lactate dehydrogenase (LDH) activity and examined the mitochondrial electron transport activity in the left ventricles under BL conditions and at 3 h post reperfusion. Our results indicate that LDH activity was significantly reduced in both genotypes following IR that likely indicates cellular damage with ensuing LDH leak into the extracellular compartment following IR-mediated injury (Figure 3). 

However, we did not observe any significant differences between FtH^fl/fl^ and FtH^SM22Δ/Δ^ littermates. The mitochondrial matrix enzyme citrate synthase (CS) was measured as it is frequently used an index of mitochondrial number. We found no difference in CS under any conditions consistent with no change in mitochondrial number in response to FtH deletion. In addition, we found that under homeostatic conditions mitochondrial complex-I (C-I)-related oxygen consumption rate was lower in FtH^SM22Δ/Δ^ hearts. Furthermore, in agreement with previous findings, we found that C-I activity was most susceptible to IR injury [27]. However, while the FtH^SM22Δ/Δ^ hearts displayed lower C-I activity under control conditions, there was no further decrease following IR in contrast to the control wildtype hearts. Furthermore, we did not observe any significant alterations in the context of other mitochondrial complex activities under BL, and IR conditions in hearts with FtH deficiency (Figure 3). 

### 2.3. Functional Significance of FtH in Myocardial IR

**Figure 4:**
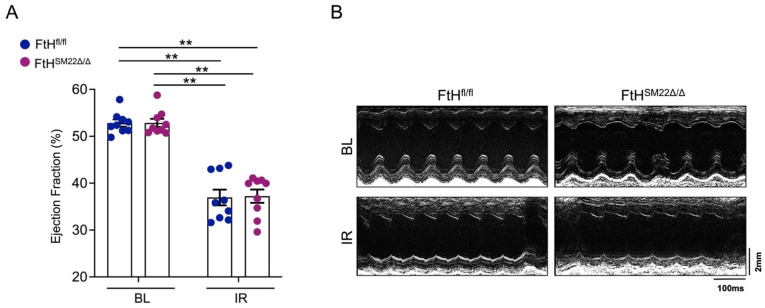
Functional significance of myocardial FtH deletion in IR on day one post IR. (**A**,**B**) FtH^fl/fl^ and FtH^SM22Δ/Δ^ mice were subjected to myocardial IR (45 min via ligation of the left anterior descending coronary artery) and left ventricular ejection fraction was measured using 2D tracing in the parasternal long-axis views. (**A**) Demonstration of ejection fraction % from FtH^fl/fl^ and FtH^SM22Δ/Δ^ mice under BL conditions and at 24 h post reperfusion. Data are presented as means ± SEM. n = 10/condition/genotype. ** *p* < 0.01. (**B**) Representative echocardiograms measured at BL and IR conditions.

The aforementioned results suggested that FtH deficiency may not necessarily lead to worsening cardiac function following IR. Accordingly, we tested left ventricular ejection fraction (EF) via ECHO analysis to investigate the functional significance of FtH in cardiac IR injury. We demonstrate that, irrespective of genotype, both FtH^fl/fl^ and FtH^SM22Δ/Δ^ littermates had substantially reduced EF on day 1 post IR (Figure 4A,B). 

**Figure 5:**
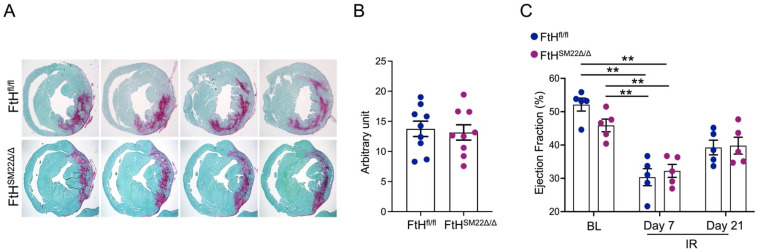
FtH deletion does not impact myocardial fibrosis or left ventricular ejection fraction. (**A**) Representative images of series of transverse sections at 7 days post IR in FtH^fl/fl^ and FtH^SM22Δ/Δ^ mice. Sirius red/fast green collagen staining marks myocardium (green) and scar (red). Magnification 4×. (**B**) Quantification of collagen deposition by measuring intensity of sirius red stain encompassing positive staining areas above threshold at seven days post IR. Data are presented as means ± SEM. (**C**) Demonstration of left ventricular ejection fraction % under BL conditions, and on days 7 and 21 post IR. Data are presented as means ± SEM. n = 5/genotype. ** *p* < 0.01.

These findings indicate that the acute decline in cardiac function was independent of myocardial FtH expression. These observations prompted us to ask whether FtH deficiency may impact the fibrosis and development of heart failure. To address this subject, we stained serial sections of myocardium to visualize and quantify collagen deposition on day 7 following IR. As illustrated in Figure 5A, despite a marked increment in collagen formation surrounding the areas that are supplied by LAD, we did not observe any differences between FtH^fl/fl^ and FtH^SM22Δ/Δ^ mice. This statement is supported by quantification of collagen deposition as illustrated in Figure 5B. These findings were further validated when we measured the EF on days 7 and 21 post IR. Similarly, while both genotypes revealed a reduction in EF at both timepoints, FtH deficiency did not alter the course of cardiac function recovery (Figure 5C).

### 2.4. Elucidation of Adaptive Mechanisms That Counteract Injurious Effects of FtH Deficiency

Despite the well-established anti-oxidant and protective attributes of FtH expression, its deletion did not appear to augment mitochondrial malfunction or worsen cardiac function following IR-induced injury. Hence, we reasoned that unbiased transcriptomic analysis may reveal potential adaptive pathways that neutralize and protect the myocardium in the absence of FtH expression. 

**Figure 6:**
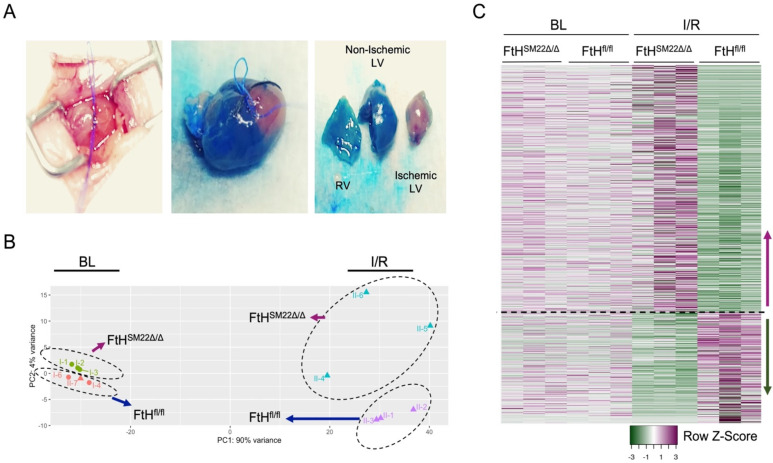
Alteration of gene expression profile in FtH^fl/fl^ and FtH^SM22Δ/Δ^ hearts following IR. (**A**) Demonstrative image illustrating dissection of the ischemic region at 3 h post reperfusion. (**B**) Principal component analysis plot of samples obtained under BL and IR conditions demonstrating clusters of samples based on their gene expression profile. (**C**) RNA isolated from the left ventricle and ischemic regions of FtH^fl/fl^ and FtH^SM22Δ/Δ^ hearts was subjected to bulk RNA-sequencing analysis. The image represents hierarchical clustering of genes that are significantly altered (fold change > 2, *p* < 0.05) between isolated myocardium from different genotypes at 3 h post reperfusion. Heatmap displays z-transformed expression values. n = 3/group.

**Figure 7:**
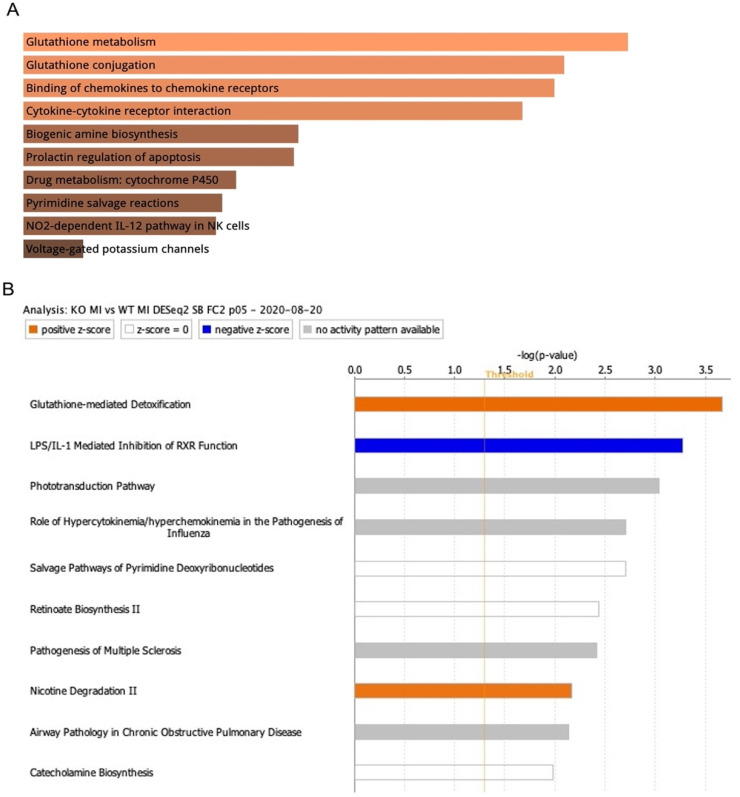
Glutathione metabolism pathway modulation following IR is accentuated in response to FtH deficiency. KEGG analyses of RNA-sequencing data showing the top 10 enriched pathways using (**A**) Enrichr platform and (**B**) Ingenuity pathways analysis platform between FtH^fl/fl^ and FtH^SM22Δ/Δ^ samples. Both analyses identify the glutathione metabolism pathway as the top canonical pathway differently modulated between genotypes in response to IR. n = 3/group.

To provide a comprehensive analysis of the FtH-mediated affected pathways, ischemic regions were dissected at three hours post reperfusion (Figure 6A) and isolated RNA was subjected to bulk RNA-sequencing using left ventricles of baseline conditions as controls. Overall, we identified 986 genes that were significantly modulated (fold > 2, *p* < 0.05) between FtH^fl/fl^ and FtH^SM22Δ/Δ^ hearts following I/R that are illustrated in the heatmap shown in Figure 6C. Using Enrichr (Figure 7A), a web-based tool for intuitive enrichment analysis [28], and Ingenuity pathway analysis software (Figure 7B) we analyzed various pathways modulated by FtH deletion in an unbiased manner. Importantly, irrespective of the form of analysis (Enrichr vs. Ingenuity Pathway software), we found that the top-rated canonical pathway that was affected by FtH deletion during IR injury was related to glutathione metabolism. 

### 2.5. HO-1 Overexpression Protects the Myocardium via Upregulation of Several Anti-Ferroptotic Genes, including Slc7a11

**Figure 8:**
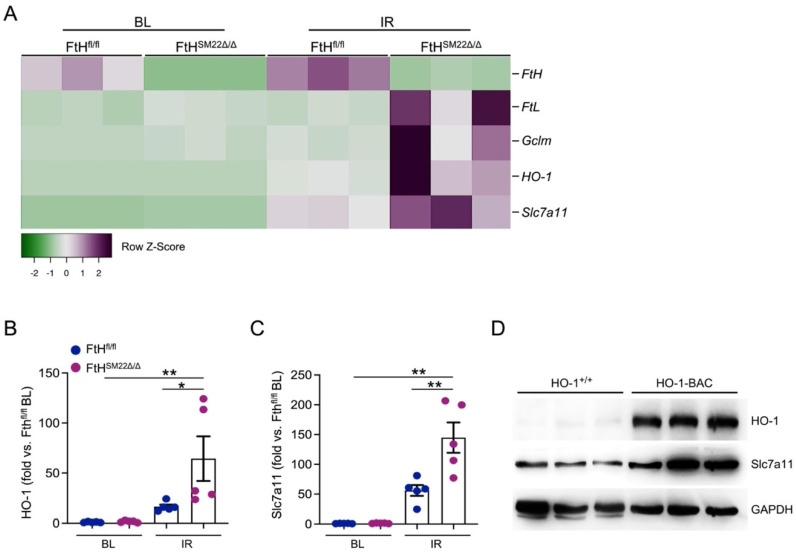
Heme oxygenase-1 (HO-1) counteracts FtH deficiency by upregulating the antiporter critical for minimizing ferroptosis, Slc7a11. (**A**) Heatmap demonstrating genes with anti-ferroptotic properties that are differentially expressed in FtH^SM22Δ/Δ^ hearts following IR compared to FtH^fl/fl^ following IR. Results was extracted from RNA-sequencing data under BL and following IR conditions from both genotypes. The differentially expressed genes were identified based on absolute fold change of two or more and adjusted *p* < 0.05. Note the marked upregulation of Slc7a11 and HO-1 in FtH^SM22Δ/Δ^ hearts in response to IR. (**B**,**C**) Results of RNA-sequencing were independently verified for HO-1 (**B**) and Slc7a11 (**C**) using RT-PCR. * *p* < 0.05 and ** *p* < 0.01. n = 5/genotype/condition. (**D**) Western blot analysis of HO-1 wildtype (HO-1^+/+^) and HO-1 overexpressing (HO-1 BAC) hearts under homeostatic conditions illustrating markedly higher levels of HO-1 and Slc7a11 expression in HO-1 BAC mice compared to controls (HO-1^+/+^). GAPDH was used as loading control.

As glutathione metabolism is central to instigation and propagation of ferroptosis, we analyzed a number of genes that are directly involved in ferroptosis. As illustrated in the heatmap (Figure 8A), myocardial deletion of FtH is coupled with significant upregulation of genes that are known to mitigate ferroptosis via various mechanisms [29]. These genes include FtL, Gclm1, HO-1, and Slc7a11 and are regulated by the Nrf2/Keap1 system [30,31]. Significant upregulation of HO-1 and Slc7a11 in FtH^SM22Δ/Δ^ hearts following injury, and in comparison to samples obtained from FtH^fl/fl^ hearts, was independently verified by RT-PCR (Figure 8B,C). Constructed on these results and the significant body of evidence supporting beneficial properties of HO-1 induction, we reasoned that upregulation of HO-1 may modulate Slc7a11 upregulation and ensuing intracellular cysteine transport, glutathione repletion, and prevention of ferroptosis. To test this premise, we used transgenic mice with global HO-1 overexpression that have been previously described and characterized [32]. As shown in Figure 8D, we corroborate that HO-1 overexpression in HO-1 BAC hearts is associated with substantially higher expression levels of Slc7a11.

## 3. Discussion

In this study, we examined whether deletion of FtH in myocardium disrupts mitochondrial biogenesis and ensuing cardiac dysfunction in a model of IR. We validated FtH deletion in cardiomyocytes via RT-PCR and immunohistochemistry. Given the high proportion of the FtH chain in myocardium, its cardinal role in minimizing participation of iron in the generation of ROS and the ferroptosis pathway, we reasoned that FtH deletion may lead to perturbation in mitochondrial homeostasis and subsequent decline in cardiac function. Our results demonstrate that FtH deletion did not impact mitochondrial surveillance processes (fission and fusion), and biogenesis under BL and IR condition. Additionally, our results indicate that myocardial FtH deletion does not heighten cardiac function decline following IR during acute phase (day 1) or recovery phase (days 7 and 21). Further analysis using unbiased transcriptomics revealed that FtH deletion leads to upregulation of a number of anti-ferroptotic genes [29], including HO-1. Our findings propose the mechanistic rationale that HO-1 overexpression balances and counteracts myocardial FtH deletion via upregulation of anti-ferroptotic genes, namely Slc7a11.

The induction of the HO-1/ferritin system has been shown to be protective in a number of injury models, including myocardial IR [33,34]. HO-1 expression is markedly induced in porcine myocardium following IR [35]. In contrast, absence of HO-1 leads to severe right ventricular dilation and infarction when mice are subjected to chronic hypoxia [36]. Cardiac-specific overexpression of HO-1 in mice is associated with significantly smaller areas of infarction following IR as well as reduced inflammation and oxidative damage [37]. Moreover, contractile function of the myocardium following ischemia is preserved in these mice in an HO-1 dose-dependent manner. Additionally, the cardioprotective effects of HO-1 induced by gene transfer have been shown to be present even one year after the initial gene transfer [38]. The role of HO-1 in doxorubicin-induced mitochondrial damage and heart failure has also been examined [39]. Results of this study demonstrate that HO-1 protects against dilated cardiomyopathy, cardiac cytoarchitectural derangement, and inflammation. Furthermore, HO-1 overexpression abrogated dilation of the sarcoplasmic reticulum as well as mitochondrial disorganization [39]. Another interesting, while anticipated, finding in this study was the compensatory upregulation in FtL that occurs in the myocardium with FtH deficiency. Compared to FtH, there is less available literature about potential cytoprotective effects of FtL. However, we recently reported that FtL possesses immunomodulatory properties and high levels of circulating FtL conferred protection against mortality and end-organ damage in a model of sepsis [40]. In support of these findings, another study found that FtL upregulation exerts an anti-inflammatory response and reduces pro-inflammatory cytokines in vitro [41].

Mitochondria are dynamic organelles that continually change their shape, by undergoing fission to generate fragmented and disconnected mitochondria (required for cell division and for removal of damaged mitochondria by mitophagy), and fusion to generate an elongated interconnected phenotype (required to replace damaged DNA and maintain normal mitochondrial respiratory function) [42]. The high iron content of mitochondrial electron transport proteins and the central role of intracellular FtH in iron homeostasis prompted us to hypothesize that deletion of FtH may negatively impact mitochondrial fission and fusion regulatory processes. Consistent with the central role of iron in mitochondrial electron transport proteins complex I, which contains a large number of FeS clusters, showed a lower activity on deletion of FtH. Based on our results, we speculate that the marked upregulation of several such well-established, protective, and anti-ferroptotic genes (that also maintain mitochondrial homeostasis) following injury in FtH^SM22Δ/Δ^ hearts prevents heightened mitochondrial damage and establishes a similar degree of perturbation/injury compared to FtH^fl/fl^ hearts. While we did not observe significant differences among genotypes following injury, we also acknowledge that perturbation in these processes may manifest in a more chronic manner and needs to be investigated in future studies. 

Iron metabolism and ferroptosis are increasingly recognized as potential novel targets to mitigate the burden of cardiovascular diseases [12,13]. Ferroptosis, as a distinct and novel form of the regulated cell-death pathway was first recognized using a new compound, erastin, with a selective lethal effect on RAS-expressing cancer cells [43]. Thanks to a growing body of evidence, multiple instigators and inhibitors of this process have now been identified with the central premise of iron metabolism [44]. The paramount role of Slc7a11 in this context is also well recognized. Slc7a11 is a cystine-glutamate antiporter that imports cystine into the cells while exporting glutamate in a 1:1 ratio [10]. Cystine is reduced to cysteine and then used as a building block during the biosynthesis of glutathione, which is a substrate for a family of antioxidant enzymes including the glutathione peroxidase 4 (GPX4) enzyme. Importantly GPX4 is well recognized as a major endogenous mechanism to suppress lipid peroxidation [45]. Moreover, inhibition of Slc7a11 expression or activity decreases cellular antioxidant capacity, leads to accumulation of reactive lipid species, and subsequently results in oxidative damage and ferroptosis [46].

Recently, it was shown that mice with targeted deletion of FtH in cardiomyocytes were susceptible to severe cardiac injury and hypertrophic cardiomyopathy in a model of iron overload state [47]. Such injury was associated with molecular signature typical of ferroptosis, including decreased glutathione levels and increased lipid peroxidation. Notably, our observations do not fully align with these reported findings. Based on our results, we reason that using different models of injury (iron overload vs. IR) may explain the discrepant results. HO-1 has previously been reported to exert anti-ferroptotic properties [48,49,50,51]. Here, in corroboration of our previous findings in a model of kidney disease [18], we report that FtH deficiency leads to marked upregulation of HO-1 following injury. While several anti-ferroptotic genes are upregulated via the Nrf2/Keap system, our results suggest that HO-1 may be the predominant factor in upregulation of such genes, particularly Slc7a11. This premise is based on previous reports outlining the HO-1 gene regulation differences between mouse and human [52]. It has been shown that the human HO-1 gene requires an internal enhancer which does not have Nrf2 sequences [52]. Furthermore, Nrf2 or Bach1 do not directly bind to the promoter or enhancer for human HO-1 and Nrf2-deficient cells are capable of upregulating the human HO-1 gene in response to nitrolipids [53]. Here, we used the HO-1 BAC mice that contain the entire human HO-1 gene along with all its regulatory regions. Using HO-1 BAC mice, we have previously shown that several transcription factors such as USF1/2, JunB, Sp1, and CTCF associate with regulatory regions of the human HO-1 gene in the kidney [32]. As shown in Figure 8D, and previously described [32], the global and constitutive overexpression of HO-1 in these mice under homeostatic conditions is suggestive of mechanisms other than Nrf2 mediating the upregulation of Slc7a11. Our results, for the first time, identify a potential novel mechanism through which beneficial properties of HO-1 may mitigate ferroptosis, namely via upregulation of Slc7a11. Nonetheless, we also propose that other mechanisms may be contributing to neutralizing FtH deficiency, such as reduced intracellular iron levels [47], that merit further investigations. Such studies would also unequivocally address the involvement of the Nrf2/Keap system in this process.

Taken together, we demonstrate that myocardial FtH deficiency does not impact IR-mediated perturbations in mitochondrial bioenergetics, fission and fusion gene expression at the transcript level, and overall cardiac function. We suggest that upregulation of several anti-ferroptotic genes may be counteracting FtH deficiency. Furthermore, our findings suggest a novel mechanistic insight by suggesting that HO-1 upregulation may inhibit the ferroptotic pathway via induction of Slc7a11 and replenishment of intracellular glutathione. 

## 4. Materials and Methods

### 4.1. Animals

FtH deletion was achieved by crossing FtH-floxed mice (FtH^fl/fl^) mice with SM22-Cre mice that express the Cre protein in smooth muscle cells and cardiomyocytes during the embryonic period [23]. This approach was used to generate mice with myocardial FtH deletion (FtH^SM22Δ/Δ^). As part of our initial characterization, we confirmed deletion of FtH in myocardium of both male and female FtH^SM22Δ/Δ^ mice. Irrespective of gender, we did not observe any major differences in terms of FtH expression in any other major organ tested. In this manuscript, we only presented data that confirms deletion of myocardial FtH in male mice as all experiments were performed in male animals. Heme oxygenase-1 (HO-1) BAC mice were generously provided by Dr. Anupam Agarwal. Mice were maintained in a sterile, controlled environment. 

### 4.2. Ischemia-Reperfusion (IR) Injury

For IR surgeries, 8–12-week-old male mice were used. All mice were anesthetized with 2% isoflurane and placed in a supine position on a heating pad (37 °C). Animals were intubated with a 19G stump needle and ventilated with room air using a MiniVent mouse ventilator (Hugo Sachs Elektronik; [Grünstraße, Germany] stroke volume 250 μL, respiratory rate 150–200 breaths per minute). Following left thoracotomy between the 2nd and 3rd ribs, the left anterior descending coronary artery (LAD) was visualized under a microscope and ligated using a 6–0 prolene suture. Regional ischemia was confirmed by visual inspection under a dissecting microscope (Leica, Deerfield, IL, USA) by discoloration of the occluded distal myocardium. For reperfusion, the ligation was released after 45 min of ischemia and the tissue allowed to re-perfuse as confirmed by visual inspection. Identification and dissection of the ischemic region was achieved by using phthalocyanine blue pigment as previously described [54]. Briefly, mice were subjected to 3 h of reperfusion after 45 min of ischemia with the released suture around the LAD left inside. Then, the mice were sacrificed. The aorta was cannulated, and the heart was washed with PBS. Subsequently, the suture around the LAD was ligated. The heart was perfused with a 5% solution of phthalo blue dye (Sigma, St. Louis, MO, USA) in normal saline (1 mL per heart). The ischemic zone and the non-ischemic zone were separated using scissors to cut along the margin of the blue dye (unstained area is the ischemic zone and the blue-dye-stained area is the non-ischemic zone).

### 4.3. Echocardiography

Transthoracic echocardiography was performed under baseline conditions and on days 1, 7, and 21 following IR injury. Briefly, mice were anesthetized with isoflurane at 1.5%, and the parasternal long axis and short axis views were taken using a FUJI Film Vevo 3100 echocardiogram machine. The LVEF was measured using 2D tracing in the parasternal long-axis views.

### 4.4. Quantification of mRNA Expression

Total RNA was isolated from cells or tissues by TRIzol (Invitrogen, Carlsbad, CA, USA) and SYBR-Green-based real-time PCR was performed on cDNA product generated from total RNA (Qiagen, Germantown, MD, USA). Relative mRNA expression was quantified using the ΔΔCt method and normalized to GAPDH mRNA as an internal control. See Table 1 for real-time PCR (RT-PCR) primers used. All reactions were performed in triplicate and specificity was monitored using melting curve analysis.

### 4.5. Western Blot Analysis

Harvested tissues were homogenized in RIPA buffer (50 mmol/L Tris-HCl, 1% NP-40, 0.25% deoxycholic acid, 150 mmol/L NaCl, 1 mmol/L EGTA, 1 mmol/L sodium orthovanadate, and 1 mmol/L sodium fluoride) with the addition of protease inhibitor (Sigma, St. Louis, MO, USA) and phosphatase inhibitor cocktail (Bimake, Houston, TX, USA). Lysates were centrifuged at 12,000 RPM for 15 min at 4 °C and supernatant was collected. Total protein was quantified by BCA protein assay (Fisher Scientific, Waltham, MA, USA) and loaded on a 12% Tris-glycine sodium dodecyl sulfate polyacrylamide electrophoresis gel at a concentration of 40 µg and transferred to an Immobilon-P PVDF membrane via electroblotting (Millipore-Sigma, St. Louis, MO, USA). Following transfer, membranes were incubated in 6% non-fat dry milk in PBS with 0.1% Tween-20 (Fisher Scientific, Waltham, MA, USA) for 1 h at room temperature and incubated overnight at 4 °C in 1–5% non-fat dry milk with mouse FtH (Santa Cruz Biotechnologies, Dallas, TX, USA, 1:1000), goat FtL (Invitrogen, Waltham, MA, USA, 1:2000), rabbit Slc7a11 (Abcam, Waltham, MA, USA, 1:1000), rabbit HO-1 (Enzo, Farmingdale, NY, USA, 1:5000), and mouse GAPDH (Sigma, St. Louis, MO, USA, 1:10,000). Membranes were incubated for 1 h at room temp with HRP-conjugated anti-mouse, or anti-rabbit secondary antibodies (Kindle Biosciences LLC, Greenwich, CT, USA). Membranes were imaged for peroxidase activity of target proteins using a KwikQuant digital imaging system with enhanced chemi-luminescence substrate (Kindle Biosciences LLC). Analysis of densitometry was performed using Licor Image Studio Lite (version 5.2, produced by LI-COR Biosciences, Lincoln, NE, USA) and normalized to GAPDH expression.

### 4.6. Immunohistochemistry

Kidneys were fixed in 10% neutral buffered formalin for 24 h and then embedded in paraffin. Paraffin-embedded 5 μm kidney sections were deparaffanized in xylenes, rehydrated in a series of ethanol rinses from 100 to 70% ethanol, then washed in distilled water. Antigen retrieval was performed in Trilogy (Cell marque) at 95 °C for 30 min. Sections were allowed to cool slowly, washed in distilled water, and incubated in 3% H_2_O_2_ for 20 min. Sections were blocked in blocking buffer containing 5% of the secondary host’s serum at room temperature for 1 h. Primary antibodies were diluted in the blocking buffer for mouse FtH (Santa Cruz Biotechnologies, Dallas, TX, USA, 1:250), goat FtL (Invitrogen, Waltham, MA, USA, 1:500), and added to sections overnight at 4 °C. Sections were washed 3 times with PBST for 5 min each. Goat anti-rabbit and donkey anti-goat secondary antibodies (Jackson ImmunoResearch Laboratories, West Grove, PA, USA, 1:500) were diluted in blocking buffer and added to the sections for 1 h at room temperature. Sections were washed 3 times with PBST for 5 min each. Chromagen substrates were mixed per the manufacturer’s instructions (Vector Labs, Burlingame, CA, USA) and added to sections. Sections were washed in distilled water, dehydrated, and mounted using xylene mounting media (PROTOCOL). All images were acquired on a BZ-X700 All-In-One Fluorescence Microscope (Keyence, Istasca, IL, USA).

### 4.7. Fast Green/Red Sirius Stain

At seven days post IR, mouse hearts were dissected out, rinsed in cold PBS, and hearts were sectioned from the apex to base at an interval of ~2 mm. Heart sections were then fixed in 10% neutral buffered formalin for 24 h and then embedded in paraffin and Fast green/Red Sirius stain was performed as previously described [55]. Percent area positive and intensity (integrated density) of Sirius red stain, as a measure of collagen deposition and fibrosis, was measured using FIJI version 1.51 n. Color, brightness, and contrast adjustments were made in an identical manner for matched sections. 

### 4.8. Measurement of Mitochondrial Respiratory Complex Activity

This procedure was performed as previously described [56,57]. Briefly, mice heart tissues from were flash frozen in liquid nitrogen. Frozen heart tissues were pulverized in a liquid nitrogen mini mortar and then mechanically homogenized with 20 strokes in a glass–glass Dounce homogenizer with MAS buffer (70 mM sucrose, 220 mM mannitol, 5 mM KH_2_PO_4_, 5 mM MgCl_2_, 1 mM EGTA, 2 mM HEPES pH 7.4). All homogenates were centrifuged at 200× *g* for 10 min at 4 °C followed by the collection of the supernatant. Protein concentration was determined using a BCA protein assay (Thermo Fisher, Waltham, MA, USA). The heart homogenates (1 µg protein) were loaded into Seahorse XF96 microplates in 20 µL of MAS buffer. The loaded plate was centrifuged at 2000× *g* for 20 min at 4 °C (no brake), then 160 µL of MAS containing cytochrome *c* (10 µM) and alamethicin (10 µg/mL) were added per well. To determine specific complex activity, the following concentration of substrates and inhibitors were used: Complex-I: NADH (1 mM) followed by rotenone (1 µM); Complex-II: succinate + rotenone (5 mM + 1 µM), followed by antimycin A (10 µM); Complex-III: duroquinol (0.5 mM) followed by antimycin A (10 µM); Complex-IV: Tetamethylphenylenediamine (TMPD) + ascorbic acid (0.5 mM + 2 mM), followed by azide (20 mM).

### 4.9. Measurement of Citrate Synthase and Lactate Dehydrogenase (LDH) Activity

Citrate synthase activity was measured spectrophotometrically as previously described (Redmann et al., 2017; Oshima et al., 2020). Briefly, the change in absorbance of 5,5′-dithiobis(2,4-nitrobenzoic acid) (DTNB) was followed after the addition of heart homogenates (5 μg) in the presence of 100 mM Tris/0.1% Triton X-100, (pH 8.0 at 37 °C), and 10 mM acetyl-CoA. Reactions were initiated by the addition of oxaloacetate (20 mM). Specific activity was calculated as the rate change normalized to total protein. For measurement of LDH activity, homogenates (10 μg) were used to measure LDH activity spectrophotometrically by monitoring the rate of oxidation of NADH at 340 nm over time in the presence of 10 mM pyruvate. Reactions were performed at 37 °C in PBS, pH 7.4 containing 0.1% Triton x-100.

### 4.10. RNA Sequencing Data Processing and Analysis

STAR (version 2.7.5a) was used to align the raw RNA-Seq fastq reads to the mouse reference genome (GRCm38 p6, Release M24) from Gencode [58]. Following alignment, HTSeq-count (version 0.11.3) was used to count the number of reads mapping to each gene [59]. Normalization and differential expression was then applied to the count files using DESeq2 [60].

### 4.11. Statistics

Statistical analyses were performed using GraphPad Prism (version 9, developed by GraphPad Software, Inc., San Diego, CA, USA) and data are presented as mean ± SEM. An unpaired 2-tailed Student’s t test was used for comparisons between 2 groups. For comparisons involving more than 2 groups, ANOVA and Newman–Keuls test were used. A *p* value less than 0.05 was considered significant. All experiments were performed at least 3 times. To avoid observer bias, the operators were blinded during experimental procedures involved in IR surgeries, echocardiography, histology, experiments involving mitochondrial biogenesis, and RNA sequencing. 

## Figures and Tables

**Table 1:** Primers for real-time PCR analysis.

PGC	Forward	5′-AGCCGTGACCACTGACAACGAG-3′
PGC	Reverse	5′-GCTGCATGGTTCTGAGTGCTAAG-3′
Drp1	Forward	5′-GGAACCAACAACAGGCAACT-3′
Drp1	Reverse	5′-GCAACTGGAACTGGCACAT-3′
Fis1	Forward	5′-AAGTATGTGCGAGGGCTGTT-3′
Fis1	Reverse	5′-AGCCAGTCCAATGAGTCCAG-3′
Opa1	Forward	5′-ATCCTAACGCCATCATCCTG-3′
Opa1	Reverse	5′-GTTGTATCCTGCTTGGACTGG-3′
Mfn1	Forward	5′-TCAGAGCCCATCTTTCAGGT-3′
Mfn1	Reverse	5′-GTTTCCAGCCCACTGTTTTC-3′
Mfn2	Forward	5′-CTCCATCAGGACGAGCAGTT-3′
Mfn2	Reverse	5′-GCACAAACACATCAGCATCC-3′
GAPDH	Forward	5′-ATCATCCCTGCATCCACT-3′
GAPDH	Reverse	5′-ATCCACGACGGACACATT-3′

## Data Availability

The accession numbers for the RNA sequencing data reported in this paper are Gene Expression Omnibus (GEO): GSE207609.
